# Chemical Derivatization and Paper Spray Ionization Mass Spectrometry for Fast Screening of Retinoic Acid in Cosmetics

**DOI:** 10.3390/molecules29184491

**Published:** 2024-09-21

**Authors:** Yuzhang Bao, Ningzi Guo, Xiaowen Hu, Bin Di, Yang Liu, Huimin Sun

**Affiliations:** 1 National Institutes for Food and Drug Control, Beijing 102629, China; baoyzya@163.com (Y.B.); guoningzi@nifdc.org.cn (N.G.); huxiaowen@nifdc.org.cn (X.H.); 2 School of Pharmaceutical Sciences, China Pharmaceutical University, Nanjing 211100, China

**Keywords:** PSI-MS, retinoic acid, cosmetics, chemical derivatization

## Abstract

As a prescription drug, retinoic acid is listed as a banned cosmetic additive in the EU and China regulations. Currently, spectrophotometric methods, including thin-layer chromatography (TLC), high-performance liquid chromatography (HPLC), and HPLC–MS/MS, are commonly used for the determination of retinoic acid. As these conventional methods require complex pretreatment and are time-consuming, chemical derivatization combined with paper spray ionization mass spectrometry was developed for the fast detection of retinoic acid in cosmetics. N,N-dimethylpiperazine iodide (DMPI) was utilized as a derivatization reagent. Carboxylic acid in retinoic acid was derivatized to carry a positive charge and was subjected to mass spectrometry analysis. Results showed that compared with non-derivatized compounds, the detection limit was increased by about 50 times. The linearity in the range of 0.005–1 μg·mL^−1^ was good. The limit of detection (LOD) was 0.0013 μg·mL^−1^, and the limit of quantification (LOQ) was 0.0043 μg·mL^−1^. The recoveries of spiked samples were in the range of 95–105%, and the RSDs were below 5%. Derivatization and paper spray ionization MS render a quick, sensitive, and accurate method for the detection of retinoic acid in a complex matrix.

## 1. Introduction

Retinoic acid [1] is a metabolite of Vitamin A in vivo. It is a prescription drug currently used in the topical treatment of acne vulgaris [2,3,4,5], psoriasis [6,7], and ichthyosis [6,7,8,9]. Oral administration of retinoic acid has strong teratogenic effects on humans and experimental animals, including mice, rats, and hamsters. Topical application of retinoic acid to the skin shows embryotoxicity and teratogenicity in mice and rabbits in the embryo-sensitive period and may cause maternal systemic toxicity. Retinoic acid may also cause redness, swelling, and erosions on healthy human skin [10,11,12,13]. Thus, retinoic acid is prohibited in cosmetics products by Chinese Safety and Technical Standards (2015) and Regulation (EC) No 1223/2009 of the European Parliament and of the Council [14,15].

It is essential to establish rapid and sensitive methods for the detection of retinoic acid in cosmetics. Currently, thin-layer chromatography (TLC) [16,17], spectrophotometry [18,19,20,21], high-performance liquid chromatography (HPLC) [22,23,24], and HPLC with tandem mass spectrometry (HPLC–MS/MS) [25,26,27,28,29] have been reported for the determination of retinoic acid in cosmetics and pharmaceuticals. HPLC–MS/MS generally has the highest sensitivity and is widely used in screening retinoic acid in complicated matrices [25,30]. Meanwhile, these methods are often time-consuming and costly.

To reduce the pretreatment time, one solution is the adoption of ambient ionization mass spectrometry (AMS). First reported in 2004, it is a new type of mass spectrometry technology that can directly analyze samples or sample surface substances under an atmospheric pressure environment, which requires no or only a simple pretreatment. AMS realizes in-situ, real-time, environmentally friendly, and rapid detection while retaining the accuracy and sensitivity of conventional mass spectrometry. Currently, the commonly used AMS includes Desorption electrospray ionization (DESI) [31,32], Direct analysis in real-time (DART) [33,34], Low-temperature plasma (LTP) [35,36], etc.

Paper Spray Ionization Mass Spectrometry (PSI-MS) [37,38,39,40] was introduced in 2010 as a type of ambient ionization mass spectrometry (AMS). It is currently applied in pharmaceuticals [41,42,43], biological matrices [44,45,46], environmental testing [47,48,49], forensic identification [50,51,52], food testing [53,54,55], etc. In this technology, filtration paper is used as the substrate, and the substance to be tested is added dropwise onto the paper substrate. In the presence of an applied electric field, normally several kilovolts, the substance dissolved in the spray solvent moves to the tip of the paper tip, forms an electrically charged spray, and is then detected by the mass spectrometer. PSI-MS is capable of high-throughput detection of compounds and requires minimal sample preparation. PSI-MS also combines the high sensitivity and accuracy of conventional mass spectrometry and is suitable for the rapid analysis of retinoic acid in cosmetics.

**Figure 1:**
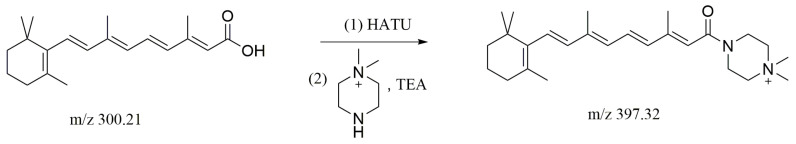
Derivative reaction of retinoic acid and DMPI.

Compounds lacking nitrogen atoms are more difficult to be protonated than nitrogen-containing compounds during PSI-MS. Adding one or more positive charges onto the original substance by chemical derivatization will greatly improve sensitivity in PSI-MS. In this study, a fast derivatization of retinoic acid with N,N-Dimethylpiperazinium iodide (DMPI) was developed, and following quantitative detection of retinoic acid derivatives in cosmetics by PSI-MS with high sensitivity was achieved (Figure 1).

## 2. Results and Discussion

### 2.1. The Selection of Derivatization Reagents

Retinoic acid is composed of only carbon, hydrogen, and oxygen. This composition results in poor ionization efficiency in the positive ion mode of PSI-MS. To enhance its sensitivity in mass spectrometry, it is necessary to introduce a positively charged moiety to retinoic acid.

Based on the functional groups within retinoic acid, carboxyl acid is derivatized. Before reacting with an amine to form an amide derivative, the carboxyl group must be activated. *O*-(7-Azabenzotriazol-1-yl)-*N*,*N*,*N’*,*N’*-tetramethyluronium hexafluorophosphate (HATU) [56], *O*-(Benzotriazol-1-yl)-*N*,*N*,*N’*,*N’*-tetramethyluronium hexafluorophosphate (HBTU), Dicyclohexylcarbodiimide (DCC), and ethyl-(*N’*,*N’*-dimethylamino)propylcarbodiimide (EDC) are all commonly used catalysts for carboxylic acid activation. Compared to other similar catalysts, HATU renders a faster reaction rate and is less susceptible to racemization. Thus, HATU was used in this study.

After activation, amines are widely used to form amides. Introducing nitrogen atoms helps the ionization process during mass spectrometry analysis. To further improve the ionization sensitivity, quarternary ammonium or piperazinium salts, which contain a positive charge, are also used as the derivatization reagent. The addition of a positive moiety to the retinoic acid will greatly improve the mass spectrometry sensitivity. Meanwhile, Guo et al. showed that the reaction between DMPI and carboxylic acid finished in less than one minute at room temperature [57]. DMPI was used in this study to quickly react with retinoic acid as the derivatization reagent. We performed mass spectrometry detection (product ion scan mode and MRM mode) on retinoic acid and its derivatives (Figures S1–S4). The response of the derivatized retinoic acid was greatly improved compared to retinoic acid without derivatization.

### 2.2. The Selection of Internal Standard

For quantitation purposes in mass spectrometry, an internal standard (IS) is often used. The structure of IS should be similar to the analyte. The isotope-labeled compound of the analyte is the most suitable IS for quantitation since the IS has similar physical and chemical properties to the analyte. However, the isotope-labeled compounds are usually expensive and not easy to obtain. Fenbufen, which has a carboxylic acid group and is structurally similar to retinoic acid, was used as the IS in the study. Therefore, Fenbufen, as the internal standard of retinoic acid, was analyzed by PSI-MS (Figures S5 and S6).

### 2.3. Optimization of Derivatization Conditions

When methanol was used as the solvent, almost no retinoic acid derivative was found. This may be caused by the reaction of methanol with the HATU-activated retinoic acid. Thus, retinoic acid, HUTA, and DMPI were dissolved in acetonitrile separately to prepare the stock solutions. The solution was stable within 240 min.

Since there are various matrices in cosmetics that may also react with HATU and DMPI, it is essential to ensure that the amounts of HATU and DMPI are in excess. It was found that the complete reaction of retinoic acid in complex matrices was achieved when the amounts of HATU and DMPI were at least 400 and 3000 times higher than the amounts of retinoic acid. Further experiments showed that the reaction was completed immediately without heating or sonication at room temperature.

### 2.4. The Optimization of Paper-Spray Ionization Mass Spectrometry Parameters

In optimizing the mass spectrometry parameters, the distance between the tip of the triangular paper and the cone of the ion source (2 mm, 4 mm, 6 mm, and 10 mm) was studied. It was found that the best response for the retinoic acid-derived compounds was obtained at a distance of 4 mm. When the paper tip is too close to the cone hole, it is easy to produce the discharge phenomenon. With the distance larger than 10 mm, the generated electrospray is almost dissipated in the environment and cannot be detected by the mass spectrometer.

**Figure 2:**
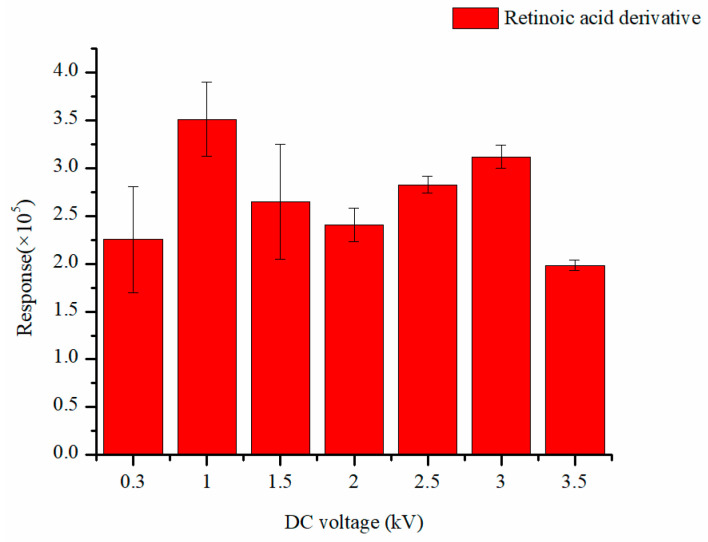
Optimization of the external DC voltage.

Various external DC voltages (0.3 kV, 1.0 kV, 1.5 kV, 2.0 kV, 2.5 kV, 3.0 kV, 3.5 kV) were also studied. The results (Figure 2) showed that the mass spectrometry response was best when the external DC voltage was 1.0 kV. This result is lower than that of the normally used 2.5–3.5 kV in the literature. This may be due to the positive charge on the retinoic acid derivative. It undergoes electrospray directly without requiring a high voltage to form charged ions first.

**Figure 3:**
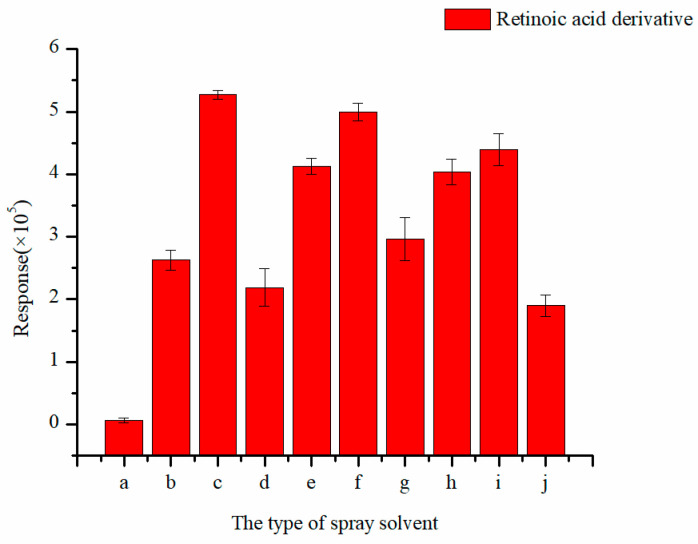
Effect of the type of spray solvent on mass spectral response (a) MeOH; (b) MeOH:H_2_O = 9:1; (c) MeOH:H_2_O = 8:2; (d) MeOH:H_2_O = 7:3; (e) ACN; (f) ACN:H_2_O = 9.5:0.5; (g) ACN:H_2_O = 8:2; (h) ACN:H_2_O = 7:3; (i) MeOH:H_2_O = 8:2 + 0.1% FA; (j) ACN:H_2_O = 9.5:0.5 + 0.1% FA.

The spray solvent serves to re-dissolve the analyte on the paper. The signal intensity is directly affected by the spray solvent. Commonly used spray solvents include water, acetonitrile, methanol, etc. As shown in Figure 3, the best response was obtained when the spray solvent was MeOH:H_2_O (*V*/*V*) = 8:2. When the spray solvent was pure methanol, the response was extremely low, probably because the retinoic acid derivative had poor solubility in methanol. When water was mixed with methanol, the solubility was improved. Interestingly, after 0.1% formic acid was added, the response was decreased. This may be due to the analyte itself being already positively charged; additional acid suppresses the ionization.

### 2.5. Linearity and Sensitivity

**Figure 4:**
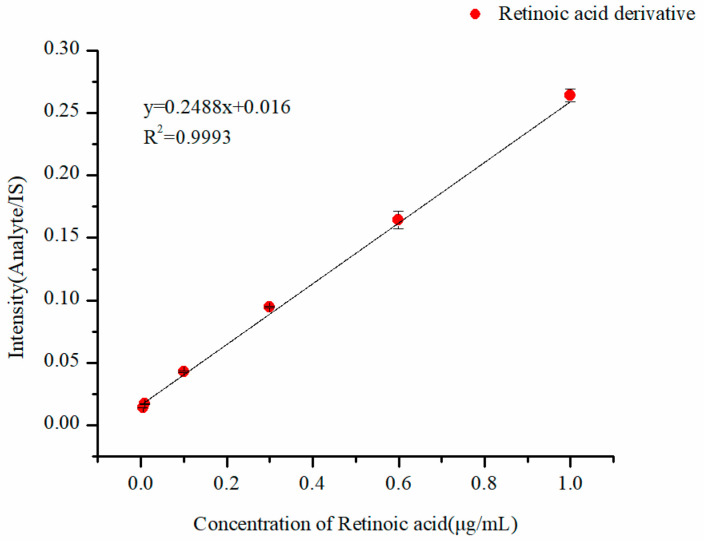
The linearity of the derivatives of retinoic acid.

Linear solutions were injected from low (0.005 µg·mL^−1^) to high (1 µg·mL^−1^) concentrations, and the injections were repeated three times for each concentration. A linear curve was plotted with the ratio of the intensity of the analyte to IS (Y) versus the concentration of retinoic acid (X) (Figure 4). The linear correlation equation was y = 0.2488x+ 0.016, and the coefficients were 0.9993. The lower limits of detection (LOD) were calculated by D = 3δ/S, where D represents LOD, δ represents the standard deviation of six injections of the blank solution, and S represents the slope of the linearity. LOD was 0.0013 µg·mL^−1^, and the LOQ was 0.0043 µg·mL^−1^.

### 2.6. The Precision of the Experiment

Retinoic acid solutions of 0.005 µg·mL^−1^, 0.01 µg·mL^−1^, 0.1 µg·mL^−1^, and 1 µg·mL^−1^ were taken and prepared according to the method described in Section 3.3. Each concentration was measured five times in parallel. The result showed that the average was calculated as 0.00496 µg·mL^−1^, 0.00986 µg·mL^−1^, 0.106 µg·mL^−1^, and 0.990 µg·mL^−1^, respectively. Their RSD were 6.87%, 6.90%, 4.77%, and 4.07%, respectively.

### 2.7. The Sample Recovery Experiment

Recovery experiments were also performed. First, 10 mg of cosmetic matrix was mixed with 5 mL linear standard solutions (0.005 µg·mL^−1^, 0.05 µg·mL^−1^ and 0.5 µg·mL^−1^), respectively. The solution was sonicated for 1 min. After 50 μL of the resulting solution and 5 μL internal standard solution (10 μg·mL^−1^) were mixed, 20 μL HATU in acetonitrile solution (1 mg·mL^−1^) was added, and the resulting solution was vortexed for 30 s. Then, 50 µL of DMPI in acetonitrile solution (3 mg·mL^−1^) and 5 µL of TEA in acetonitrile solution (1 mol·L^−1^) were added; the final solution was vortexed for 30 s and analyzed by PSI-MS immediately.

Recoveries were tested at low (0.005 µg·mL^−1^), medium (0.05 µg·mL^−1^) and high (0.5 µg·mL^−1^) concentrations. Each concentration was measured three times. The average recoveries of the samples were calculated to be 102.40%, 100.79%, and 99.85%, and the RSD were all lower than 5%.

### 2.8. The Complex Matrix Sample Detection

To test the practicability of the method, retinoic acid was added to a complex matrix (cream with glycerol, caprylic/capric triglyceride, cetearyl alcohol, 1,2-pentanediol, and glyceryl stearatese) and measured using the derivatization and PSI-MS method. The results showed that (Table 1) the method was able to quantitatively determine the retinoic acid in a complex matrix.

## 3. Materials and Methods

### 3.1. The Instruments

All experiments were carried out with an Agilent 1290 HPLC coupled with a 6495 triple quadruple mass spectrometer (Palo Alto, CA, USA). Data were acquired and processed by Agilent MassHunter Workstation 10.1 (Palo Alto, CA, USA). HB-Z303-1AC high-voltage DC power supply (Tianjin Hengbo High Voltage Power Supply Factory, Tianjin, China) and KQ-500DA CNC ultrasonic cleaner (Kunshan Ultrasonic Instrument Co., Ltd., Kunshan, China) were used. Grade 1 chromatographic paper was from Whatman (Stevenage, UK).

### 3.2. Materials and Reagents

Retinoic acid and Fenbufen were from the National Institutes for Food and Drug Control (Beijing, China). Methanol was purchased from Merck (Darmstadt, Germany). Acetonitrile was purchased from Fisher Scientific (Waltham, MA, USA). Triethylamine was from Taitan Science and Technology (Shanghai, China). HATU was purchased from TCI Chemicals (Shanghai, China). All reagents were used directly without further purification. DMPI was synthesized in the laboratory following a previously published procedure [57]. The structure of DMPI was consistent with the literature [57]: ^1^H NMR (D_2_O,TMS): δ:3.41(6H,s,CH_3_), δ:3.79–3.87(8H,m,CH_2_); HRMS: C_6_H_15_N_2_^+^ (calc.: 115.1229, found: 115.1230).

### 3.3. Solution Preparation

Standard stock solution (100 μg·mL^−1^): After 10 mg retinoic acid was transferred to a 10 mL volumetric flask, 1 mL TEA in acetonitrile (1 mol·L^−1^) was added to dissolve the solid; the resulting solution was diluted to volume with acetonitrile. Then, 1 mL of the resulting solution was transferred to a 10 mL volumetric flask and was diluted to the volume with acetonitrile.

Linear standard solutions: Standard solution was diluted to 0.005, 0.01, 0.1, 0.3, 0.6, and 1 μg·mL^−1^ with acetonitrile.

Internal standard solution (10 μg·mL^−1^): After 10 mg Fenbufen was transferred to a 10 mL volumetric flask, 1 mL TEA in acetonitrile (1 mol·L^−1^) was added to dissolve the solid; the resulting solution was diluted to volume with acetonitrile. Then, 1 mL of the solution was transferred to a 100 mL volumetric flask and was diluted to the volume with acetonitrile.

### 3.4. Derivative Reaction

Derivatization of retinoic acid: After 5 µL of internal standard solution and 50 µL of linear standard solution were mixed, 20 µL of HATU in acetonitrile solution (1 mg·mL^−1^) was added to the solution, and the resulting solution was vortexed for 10 s. Then 50 µL of DMPI in acetonitrile solution (3 mg·mL^−1^) and 5 µL of TEA in acetonitrile solution (1 mol·L^−1^) were added; the final solution was vortexed for 30 s and analyzed by PSI-MS immediately.

### 3.5. Paper Spray Mass Spectrometry Parameters

An isosceles triangular chromatography paper, 5 mm long at the bottom and 15 mm high, is fixed to a steel table with copper clamps so that the tip of the paper is directed towards the hole in the cone of the ion source of the mass spectrometer. After 2 µL of the derivatization solution was added to the paper substrate, the paper was dried for 1 min. After a voltage of 1.0 kV was applied, 20 µL of the sprayed solvent was added. The derivative compounds adsorbed on the paper are dissolved in the spray solvent and brought to the tip of the paper by the applied voltage to form an electrospray, which was detected in the mass spectrometer.

**Figure 5:**
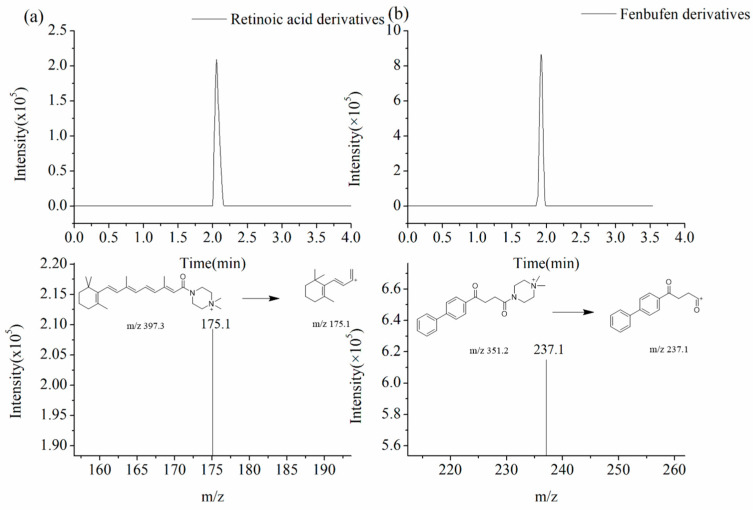
Fragmentation path of (**a**) mass spectra of retinoic acid derivative (MRM mode) and (**b**) mass spectra of Fenbufen derivative (MRM mode).

For the mass spectrometer, the collision voltage was 24 eV, and the parent ions and daughter ions were 397.3→175.1 for retinoic acid and 351.2→237.1 for Fenbufen (Figure 5).

## 4. Conclusions

In the present work, a chemical derivatization and PSI-MS method were established for fast screening of retinoic acid in cosmetics. DMPI, as a derivatization reagent, can react with retinoic acid quickly at room temperature. The reaction is not interfered with by many ingredients, such as glycerol and caprylic/capric triglyceride in cosmetics. After derivatization, the sensitivity increased more than 50 times compared to un-derivatized retinoic acid (Figure S7). Compared to the traditional HPLC–MS method, no extraction and separation of retinoic acid in cosmetics is required. This strategy can be used for fast screening of low-response analytes on PSI-MS in cosmetics.

## Figures and Tables

**Table 1:** Detection of retinoic acid in complex matrices.

Chemical Compound	Added (µg·mL^−1^)	Found (µg·mL^−1^)	Recovery Rate (%)	Average Recovery Rates (%)	RSD% (N = 3)
		0.00525	104.94		
Retinoic acid	0.005	0.00524	104.78	102.40	4.15
		0.00487	97.49		
		0.0493	98.54		
	0.05	0.0491	98.26	100.79	4.11
		0.0528	105.57		
		0.497	99.47		
	0.5	0.486	97.20	99.85	2.86
		0.514	102.87		

## Data Availability

The data presented in this study are available on request from the corresponding author.

## Supplementary Materials

The following supporting information can be downloaded at: https://www.mdpi.com/article/10.3390/molecules29184491/s1, Figure S1: Mass spectrum of retinoic acid without derivatization (Product ion scan mode); Figure S2: Mass spectrum of retinoic acid derivatives (Product ion scan mode); Figure S3: Mass spectrum of retinoic acid (MRM mode); Figure S4: Mass spectrum of retinoic acid derivatives (MRM mode); Figure S5: Mass spectrum of Fenbufen (Product ion scan mode); Figure S6: Mass spectrum of Fenbufen derivatives (Product ion scan mode); Figure S7: The linearity of the retinoic acid without derivatization.
