# Supplementary material for: Chemical Derivatization and Paper Spray Ionization Mass Spectrometry for Fast Screening of Retinoic Acid in Cosmetics
Source: Molecules. 2024 Sep 21;29(18):4491. doi: 10.3390/molecules29184491 (PMC11434060; doi:10.3390/molecules29184491)
Supplement: Supplementary file 1 [file molecules-29-04491-s001.zip › molecules-3178704-supplementary.pdf]

Supporting Information

Chemical Derivatization and Paper spray Ionization Mass Spectrometry for fast screening of Retinoic Acid in Cosmetics

Yuzhang Bao[1,2], Ningzi Guo[1], Xiaowen Hu[1], Bin Di[2], Yang Liu [1*] and Huimin Sun[1*]

1 National Institutes for Food and Drug Control, Beijing 102629, China;

2 School of Pharmaceutical Sciences, China Pharmaceutical University, Nanjing, 211100; China;

* Correspondence: yangliu@nifdc.org.cn; sunhm@126.com

Table of Content

Figure S1. Mass spectrum of retinoic acid(Product ion scan mode)…………….3

Figure S2. Mass spectrum of retinoic acid derivatives(Product ion scan mode)
…………………………………………………………………………………….3

Figure S3. Mass spectrum of retinoic acid(MRM mode)……………………..…4

Figure S4. Mass spectrum of retinoic acid derivatives(MRM mode)….……….4

Figure S5. Mass spectrum of Fenbufen(Product ion scan mode)……………….5

Figure S6. Mass spectrum of Fenbufen derivatives(Product ion scan mode)….5

Figure S7. The linearity of the retinoic acid………………………………………6

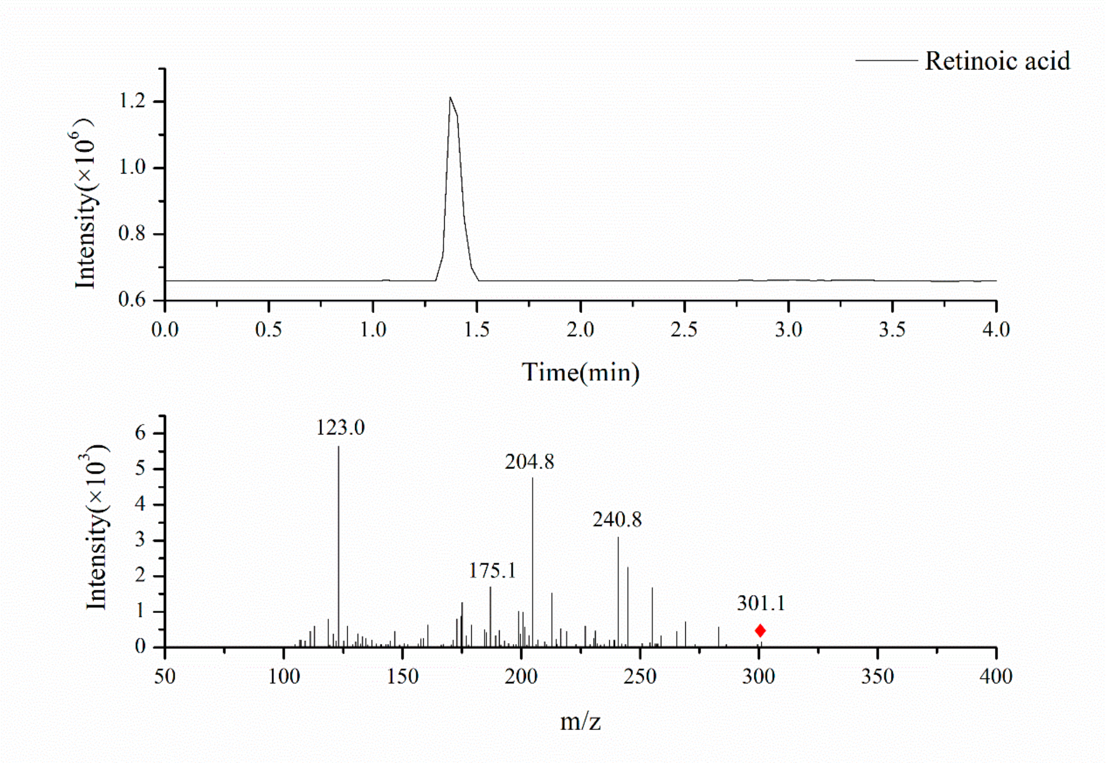

Figure S1. Mass spectrum of retinoic acid without derivatization (Product ion scan mode)

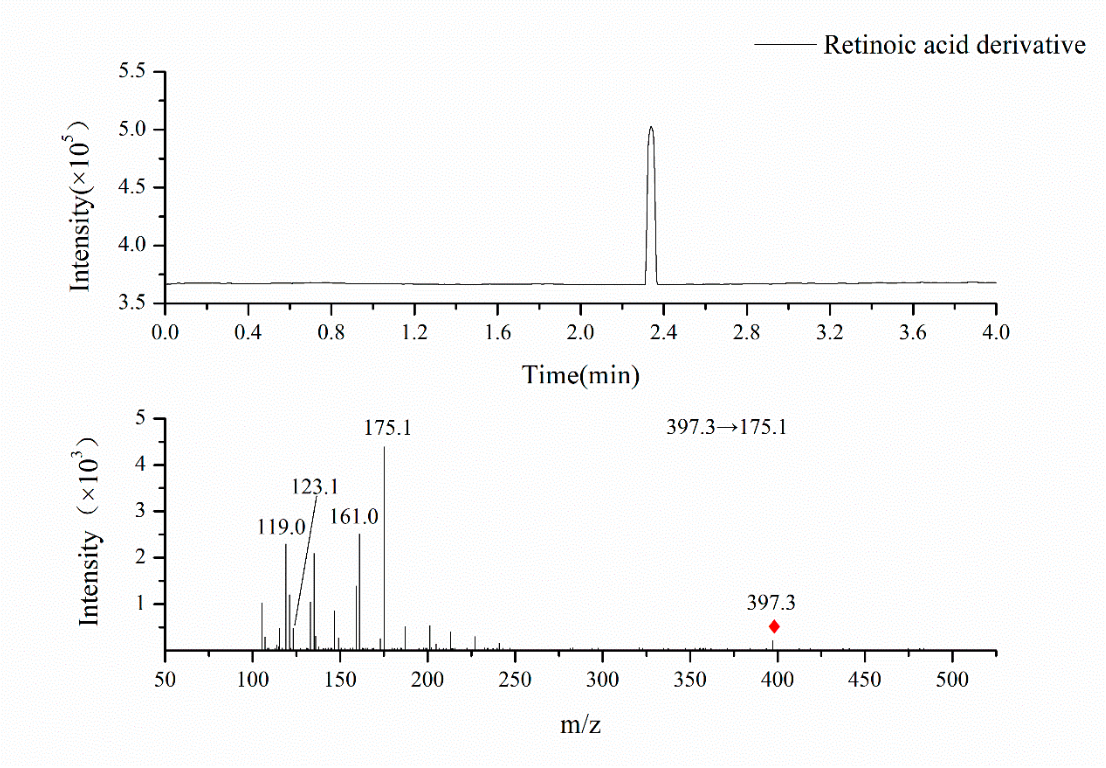

Figure S2. Mass spectrum of retinoic acid derivatives (Product ion scan mode)

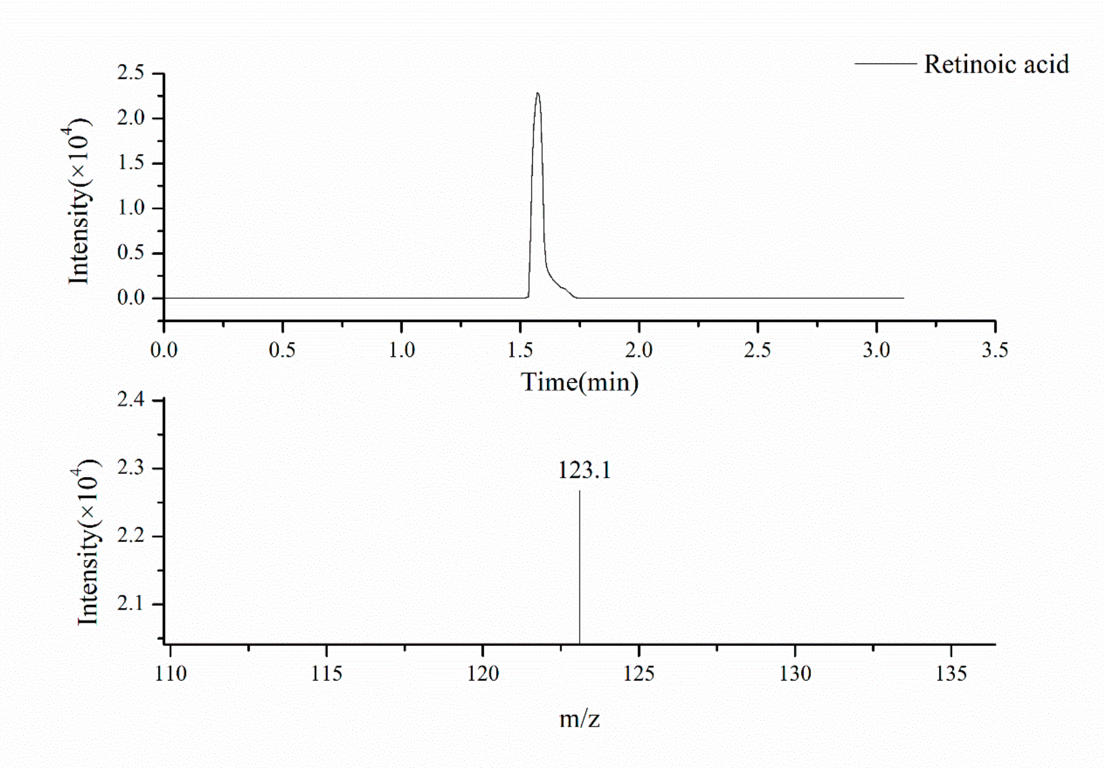

Figure S3. Mass spectrum of retinoic acid (MRM mode)

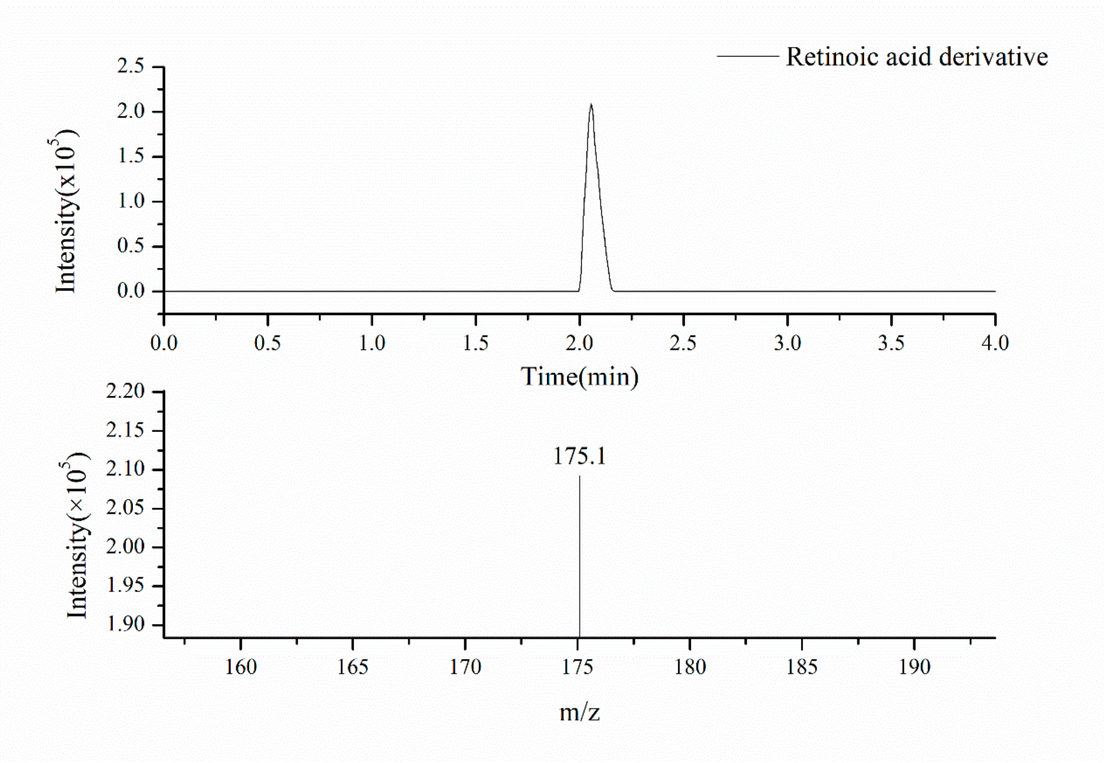

Figure S4. Mass spectrum of retinoic acid derivatives (MRM mode)

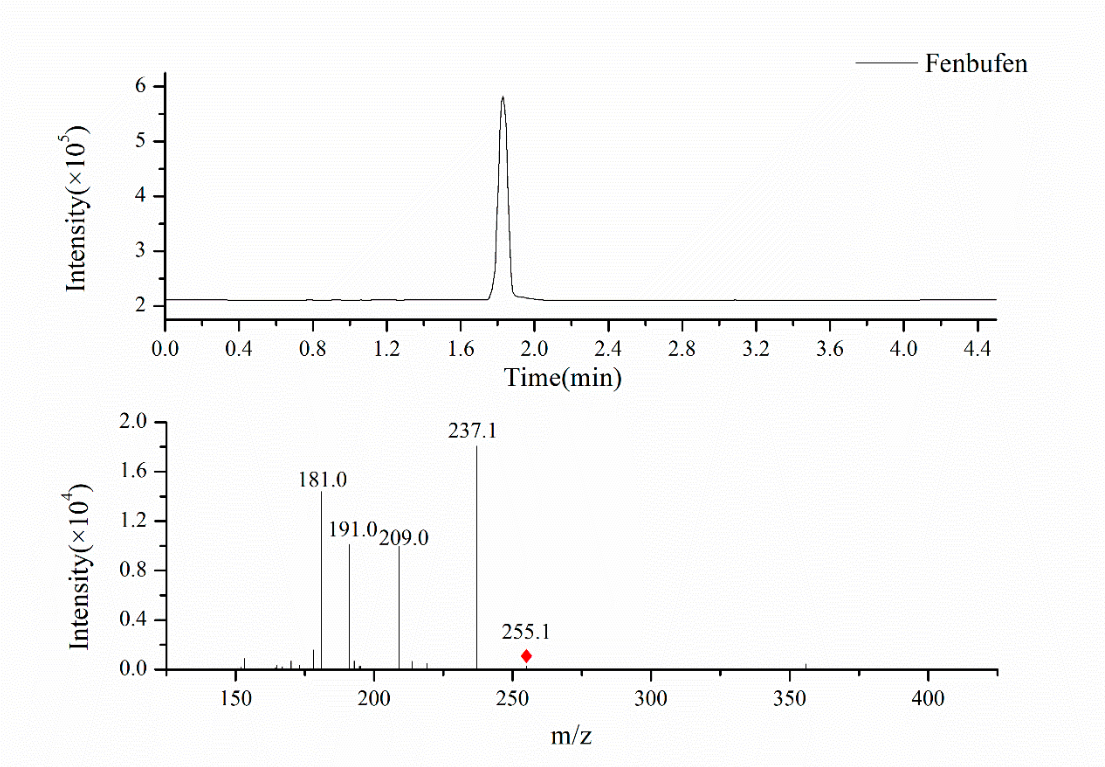

Figure S5. Mass spectrum of Fenbufen(Product ion scan mode)

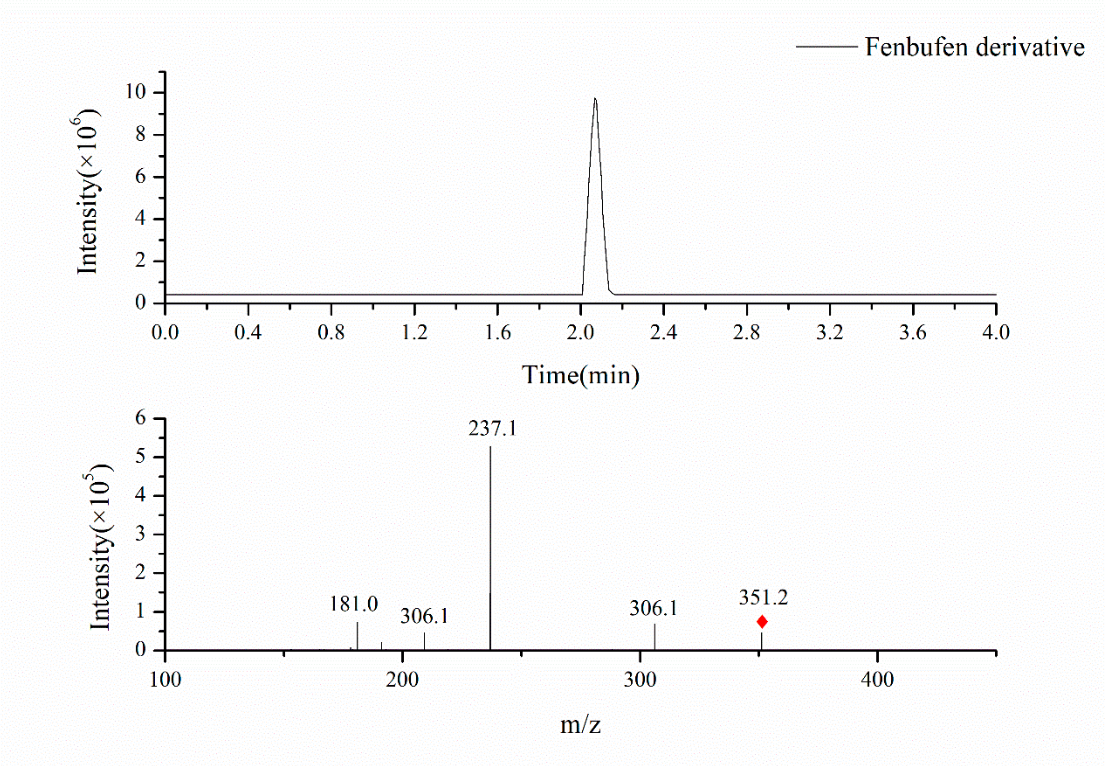

Figure S6. Mass spectrum of Fenbufen derivatives(Product ion scan mode)

Linear solutions of retinoic acid without derivatization were injected from low (0.1 μg·mL-1) to high (20 μg·mL-1) concentrations and the injections were repeated three times for each concentration. A linear curve was plotted with the ratio of the intensity of the analyte to IS (Y) versus the concentration of retinoic acid (X) (Figure S7). The linear correlation equation was y = 0.00844x+ 0.02147 and the coefficients were 0.99376. The lower limits of detection (LOD) were calculated by D = 3δ/S, where D represents LOD, δ represents the standard deviation of six injections of the blank solution and S represents the slope of the linearity. LOD was 0.071 μg·mL-1, and the LOQ was 0.237 μg·mL-1.

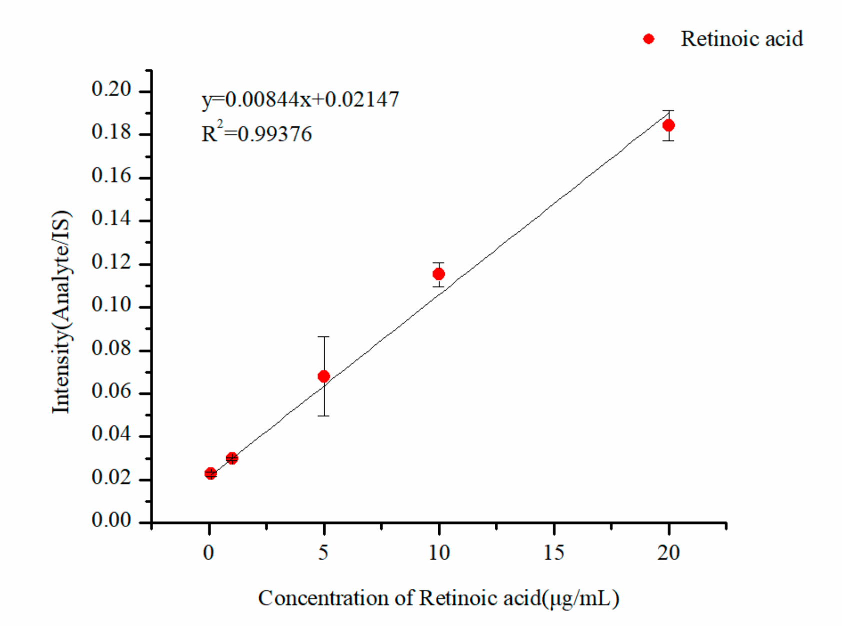

Figure S7. The linearity of the retinoic acid without derivatization
